# Radiomic Feature-Based Nomogram: A Novel Technique to Predict EGFR-Activating Mutations for EGFR Tyrosin Kinase Inhibitor Therapy

**DOI:** 10.3389/fonc.2021.590937

**Published:** 2021-08-06

**Authors:** Qiaoyou Weng, Junguo Hui, Hailin Wang, Chuanqiang Lan, Jiansheng Huang, Chun Zhao, Liyun Zheng, Shiji Fang, Minjiang Chen, Chenying Lu, Yuyan Bao, Peipei Pang, Min Xu, Weibo Mao, Zufei Wang, Jianfei Tu, Yuan Huang, Jiansong Ji

**Affiliations:** ^1^Key Laboratory of Imaging Diagnosis and Minimally Invasive Intervention Research, Lishui Hospital of Zhejiang University, Lishui, China; ^2^Department of Thoracic Surgery, Lishui Hospital of Zhejiang University, Lishui, China; ^3^Department of Pharmacy, Sanmen People’s Hospital of Zhejiang, Sanmen, China; ^4^Department of Pharmaceuticals Diagnosis, General Electric (GE) Healthcare, Hangzhou, China; ^5^Department of Pathology, Lishui Hospital of Zhejiang University, Lishui, China

**Keywords:** NSCLC, EGFR-activating mutation, clinical features, radiomics, nomogram

## Abstract

**Objectives:**

To develop and validate a radiomic feature-based nomogram for preoperative discriminating the epidermal growth factor receptor (EGFR) activating mutation from wild-type EGFR in non-small cell lung cancer (NSCLC) patients.

**Material:**

A group of 301 NSCLC patients were retrospectively reviewed. The EGFR mutation status was determined by ARMS PCR analysis. All patients underwent nonenhanced CT before surgery. Radiomic features were extracted (GE healthcare). The maximum relevance minimum redundancy (mRMR) and LASSO, were used to select features. We incorporated the independent clinical features into the radiomic feature model and formed a joint model (i.e., the radiomic feature-based nomogram). The performance of the joint model was compared with that of the other two models.

**Results:**

In total, 396 radiomic features were extracted. A radiomic signature model comprising 9 selected features was established for discriminating patients with EGFR-activating mutations from wild-type EGFR. The radiomic score (Radscore) in the two groups was significantly different between patients with wild-type EGFR and EGFR-activating mutations (training cohort: P<0.0001; validation cohort: P=0.0061). Five clinical features were retained and contributed as the clinical feature model. Compared to the radiomic feature model alone, the nomogram incorporating the clinical features and Radscore exhibited improved sensitivity and discrimination for predicting EGFR-activating mutations (sensitivity: training cohort: 0.84, validation cohort: 0.76; AUC: training cohort: 0.81, validation cohort: 0.75). Decision curve analysis demonstrated that the nomogram was clinically useful and surpassed traditional clinical and radiomic features.

**Conclusions:**

The joint model showed favorable performance in the individualized, noninvasive prediction of EGFR-activating mutations in NSCLC patients.

## Introduction

With the development of molecular biology in cancer therapy, the treatment of NSCLC patients has become increasingly based not only on the patient’s clinical characteristics and tumor morphology but also on individual mutational profiles (1). EGFR-activating mutations, including exon 19 deletion (DEL19) and exon 21 substitution (L858R), account for approximately 90% of all EGFR mutations in advanced NSCLC patients (2). For advanced NSCLC patients with EGFR-activating mutations, treatment with EGFR tyrosine kinase inhibitors (EGFR TKIs), such as gefitinib and afatinib, has become the standard of care (3, 4). Accumulating evidence suggests that EGFR TKIs can significantly prolong progression-free survival (PFS) compared to standard chemotherapy in this genetically distinct subset of patients (5, 6). Thus, the detection of EGFR-activating mutations at the time of initial diagnosis, before treatment, is critical.

Gene mutation testing can uncover pivotal information connected to underlying molecular biology. The most commonly used approach for obtaining specimens for a specific diagnosis and molecular testing is biopsy. However, the tissue acquired by invasive techniques may fail to represent the anatomic, functional, and physiological properties of cancer. Clinical studies have suggested that 10% to 20% of all NSCLC biopsies are inadequate for molecular analysis because of a lack of either sufficient tumor cells or amplifiable DNA (7). Moreover, intratumoral heterogeneity due to the diverse collection of cells harboring distinct molecular signatures will result in differential levels of sensitivity to treatment (8). Thus, an alternative approach for genetic testing is needed.

Computed tomography (CT) imaging presents a perspective of the entire tumor and its microenvironment, allowing prediction of the EGFR mutation status globally (9, 10). Radiomics refers to the computerized extraction of a large number of quantitative radiomic features from radiologic images, and this method has unique potential to reveal tumor-related information, such as pathological features, biomarker expression and genomic features, using machine learning algorithms (11, 12). Radiomics provides quantitative and objective data collected from medical images to be utilized within clinical-decision support systems to improve diagnostic, prognostic, and predictive accuracy, especially in lung cancer (13–15). Developing such a quantitative imaging technique and testing its validity may offer a new non-invasive and convenient approach for the better management of therapeutic strategies, resulting in optimized clinical and economic benefits to the patient.

Herein, we examined the correlation between 396 radiomic features and EGFR-activating mutation subtypes in two independent cohorts comprising 301 NSCLC patients. Furthermore, we created a user-friendly nomogram by incorporating the radiomic signature with the clinical characteristics to predict the probability of an event based on the individual profile of each patient. Our results reveal that the combination of the repeatable, reproducible and low-cost CT-derived radiomic signature and the clinical parameters can be used for evaluating the EGFR-activating mutation status. This may have important clinical influence, notably by allowing the better personalization of target therapy for NSCLC patients with EGFR-activating mutations.

## Materials And Methods

### Dataset

Our study was approved by the institutional review board of Lishui Hospital of Zhejiang University. Because of its retrospective nature, requirement for informed consent was waived. Patients who were diagnosed with pathologically confirmed NSCLC from June 30, 2015, to January 18, 2018, were enrolled. A total of 590 were included according to the following inclusion criteria (1): CT imaging performed within one month before surgery (2); histological diagnosis of NSCLC (3); EGFR mutations (EGFR EXON18 G719X、EGFR EXON19 19-Del、EGFR EXON20 T790M、EGFR EXON20 20-Ins、EGFR EXON20 S768I、EGFR EXON21 L858R、EGFR EXON21 L861Q) detected by amplification refractory mutation system-Scorpion real-time PCR (ARMS-PCR); and (4) clinical data were available. Thereafter, 289 patients were excluded according to the following exclusion criteria (1): preoperative treatment at the time of the initial diagnosis (n=96) (2); tissue sample obtained by biopsy rather than surgery (n=138); and (3) histological diagnosis of SCLC (n=55). Eventually, a total of 301 patients were enrolled in our study; 210 patients and 91 patients were allocated to the training and validation cohorts, respectively with a ratio of 7:3 (16).

### CT Image Acquisition and Interpretation

Patients underwent preoperative unenhanced CT scanning using a 64-channel Philips Brilliance CT system (Philips Medical Systems). Details regarding the acquisition parameters were set as follows: tube current, 200 mA; tube voltage, 120 kV; slice thickness, 0.9 mm; collimation width, 40 mm (64 × 0.625 mm); reconstruction interval with iDose3 hybrid iterative reconstruction algorithm, 0.45 mm; scan field of view (SFOV), 15-20 cm; pitch, 1.2; rotation time, 350 ms; and pixel matrix size, 1024×1024. The images were processed in the Extended Brilliance Workspace (EBW, Philips). Multi-planar reconstruction was used for image reconstruction with a thickness of 5 mm.

Two thoracic radiologists with 9 and 13 years of experience (H.W. and C.L.) performed retrospective reviews independently. Disagreements were settled by the third radiologist who had 20 years of experience (J.J.). The image features included the following (1): size and (2) volume, measured using the Extended Brilliance Workspace and Lung Nodule Assessment software (Philips) (3); lobe (4); cancer type(primary cancer or metastasis cancer) (5); tumor location (6); shape: regular (round or oval) or irregular (17) (6); lobulation (present/absent) (7); speculation (present/absent) (8); air bronchogram (present/absent) (9); necrosis (present/absent) (10); pleural retraction (present/absent) (11); calcification (present/absent); and (12) pleural effusion (present/absent).

### Tumor Segmentation and Radiomic Feature Extraction

CT images of selected patients were exported from the picture archiving and communication system (PACS) according to the inclusion and exclusion criteria. ITK-SNAP software (version 3.4.0, www.itk-snap.org) was used for three-dimensional semi-automatic segmentation (18). All images were automatically segmented and adjusted by a radiologist with 18 years of experience (Z.W., reader 1), who repeated the same procedure within 2 weeks. The interobserver reproducibility of each segmentation was evaluated by another radiologist with 20 years of clinical experience (J.J., reader 2).

Radiomic features were extracted from the ROI by commercial software Artificial Intelligence Kit (A.K) which developed by GE Healthcare (19). A total of 396 high-dimensional features were extracted from each individual, and these features were divided into 5 categories **(** **Supplementary Figure 1** **)**: histogram (n=42), form factor (n=9), grey level co-occurrence matrix (GLCM) (n=154), run-length matrix (RLM) (n=180), and grey level zone size matrix (GLZSM) (n=11).

### Inter- and Intraobserver Reproducibility

The inter- and intraobserver reproducibility of semantic image features, tumor segmentation and feature extraction were evaluated by intraclass correlation coefficients (ICCs). Two radiologists specialized in chest CT interpretation initially analyzed the images obtained from 30 randomly selected patients within 2 weeks in a blinded fashion. ICCs greater than 0.75 were considered as good consistency, and the remaining image segmentation was performed by reader 1.

### Radiomic Feature-Based Prediction Model Construction

We built the radiomic signature model based on selected features from the training cohort. Z‐score was applied to feature normalization before feature selection. Two feature selection methods, maximum relevance minimum redundancy (mRMR) and least absolute shrinkage and selection operator (LASSO), were used to select the features. First, mRMR was performed to eliminate redundant and irrelevant features. LASSO was used to select the most useful features by penalty parameter tuning and 10-fold cross-validation based on the minimum criteria. LASSO includes choosing the regular parameter λ to determine the number of features. After the number of features was determined, the most predictive subset of features was chosen, and the corresponding coefficients were evaluated. The coefficients for most radiomic features were reduced to zero, and any remaining radiomic features with non-zero coefficients were selected. Next, we built a model with selected radiomic features. A radiomic score (Radscore) was computed for each patient through a linear combination of selected features weighted by their respective coefficients. The final formula for the Radscore was as follows: “Radscore = -0.152*Small Area Emphasis + -0.097*Long Run High Grey Level Emphasis_angle0_offset4 + 0.035*Cluster Prominence _All Direction_offset7_SD + 0.082*Inverse Difference Moment_All Direction_offset4_SD + 0*Low Grey Level Run Emphasis_All Direction_offset4_SD + -0.064*Long Run Low Grey Level Emphasis_All Direction_offset7_SD + 0.275*Correlation_angle0_offset7 + 0.211*std Deviation + -0.068*GLCM Energy_All Direction_offset4_SD + -0.018”. Furthermore, the Radscore was compared between the wild-type EGFR and EGFR-activating mutations in both the training and validation cohorts.

Logistic regression with L1 regularization was performed to select the independent clinical predictors in the training cohort. Prediction models combining radiomic features and clinical variables were established. We built a radiomic nomogram based on the multivariate logistic regression model in the training cohort, and receiver operating characteristic (ROC) curves were developed to evaluate the discriminatory ability of the nomogram. The calibration curve of the nomogram was used to assess how closely the nomogram predicted EGFR-activating mutations relative to the actual probability (20, 21). The Hosmer-Lemeshow test was used to evaluate the goodness-of-fit of the calibration curve (22). In addition, decision curve analysis (DCA) was used to determine the clinical usefulness of the prediction model by quantifying the net benefits at different threshold probabilities. DCA estimates the net benefit of a model through the difference between the true-positive and false-positive rates, weighted by the odds of the selected threshold probability of risk (23).

### Statistical Analysis

Statistical analysis was performed using R software (version 3.3) for quantitative feature analysis. The characteristic features of patients with EGFR-activating mutations and wild-type EGFR were compared by Student’s t-test for normally distributed data; otherwise, the Mann-Whitney U test was used. Multivariate binary logistic regression was performed with the “rms” package. A nomogram was established by incorporating significant characteristic features and radiomic features. ROC curves were plotted to evaluate the diagnostic efficiency of the model. The area under the ROC curve (AUC) was then calculated. The nomogram was constructed and the calibration plots were created using the “rms” package. A p-value <0.05 was considered significant.

## Results

### Clinical Characteristics

A total of 301 patients were enrolled in this study, 152 patients (50.5%) were determined to have the EGFR exon 21 L858R mutation or the EGFR exon 19 DEL 19 mutation, which are both considered as EGFR-activating mutations, 149 patients (49.5%) presented with wild-type EGFR. There were 103 males and 49 females with EGFR-activating mutations and 53 males and 96 females with wild-type EGFR, respectively; the mean age was 64.95 (**Table 1**).

**Table 1:** Characteristics of 301 NSCLC patients, according to the presence of the EGFR activating mutation.

		Univariate Cox regression			Multivariate Cox regression
	Total	EGFR Activating Mutation	EGFR Wild Type	P	P
**Gender**				**<0.001**	NA
Male	156	103	53		
Female	145	49	96		
**Age**	64.95 ± 10.52	64.68 ± 10.70	65.23 ± 10.36	0.647	
**Smoking Status**				**<0.001**	**<0.0001**
Active	110	79	31		
Inactive	191	73	118		
**Size(cm)**	1.9 (2.9, 4.6)	3.15 (1.98, 5.03)	2.6 (1.8, 4.2)	0.062	
**Volume(cm^3^)**	9.08 (2.23, 30.39)	12.64 (2.78, 45.59)	6.86 (1.73, 25.50)	**0.032**	NA
**Lobe**				0.094	
Left Upper	89	39	50		
Left Middle	0	0	0		
Left Lower	54	26	28		
Right Upper	78	36	42		
Right Middle	16	10	6		
Right Lower	64	41	23		
**Cancer Type**				>0.999	
Primary Cancer	296	149	147		
Metastasis Cancer	5	3	2		
**Tumor Location**				0.393	
Peripheral	140	67	73		
Central	161	85	76		
**Concomitant other malignancy**				0.636	
Present	16	9	7		
Absent	285	143	142		
**Shape**				0.259	
Regular	36	15	21		
Irregular	265	137	128		
**Lobulated**				0.51	
Present	274	140	134		
Absent	27	12	15		
**Spiculated**				**0.021**	**0.076**
Present	199	91	108		
Absent	102	61	41		
**Air-bronchogram**				**0.014**	**0.039**
Present	80	31	49		
Absent	221	121	100		
**Necrosis**				**0.009**	NA
Present	113	68	45		
Absent	188	84	104		
**Pleural Retraction**				0.136	
Present	240	116	124		
Absent	61	36	25		
**Calcification**				0.547	
Present	35	16	19		
Absent	266	136	130		
**Pleural Effusion**				0.189	
Present	83	47	36		
Absent	218	105	113		
**CEA**				**<0.001**	**0.004**
Normal	96	1	95		
Abnormal	205	148	57		
**SCCA**				**0.006**	**0.026**
Normal	258	136	122		
Abnormal	43	13	30		
**CYFRA21-1**				**<0.001**	NA
Normal	68	1	67		
Abnormal	233	148	85		
**NSE**				**<0.001**	NA
Normal	98	3	95		
Abnormal	203	146	57		
**ProGRP**				0.952	
Normal	269	133	136		
Abnormal	32	16	16		

Age is expressed as Mean ± SD. Size, and volume are expressed as Quantiles (Q1, Q3)/Median (interquartile range). Otherwise, data are number of patients.

CEA, Carcinoembryonic antigen, SCCA, Squamous cell carcinoma antigen, CYFRA21-1, Cytokeratin 19-fragments, NSE, Neuron specific enolase, ProGRP, Progastrin-releasing peptide. The P value marked bold indicated statistical significance.

Univariate analysis revealed that sex, smoking status, tumor volume, spiculation, air bronchogram, necrosis, CEA, SCC, CYFRA21-1 and NSE were significantly associated with EGFR-activating mutations. Further multivariate analysis suggested that smoking status (OR: 5.79, 95% CI: 2.93-11.45, P<0.0001), spiculation (OR: 1.82, 95% CI: 0.94-3.51, P=0.076), air bronchogram (OR: 2.18, 95% CI 1.04-4.57, P=0.039), CEA (OR: 2.57, 95% CI: 1.35-4.87, P=0.004) and SCCA (OR: 0.37, 95% CI 0.15-0.89, P=0.026) were independent predictors of EGFR-activating mutations (**Table 1**). Satisfactory interobserver and intraobserver reproducibility of the clinical features was achieved (ICC=0.83, 0.79).

### Radiomic Signature Construction, Validation, and Evaluation

**Figure 1:**
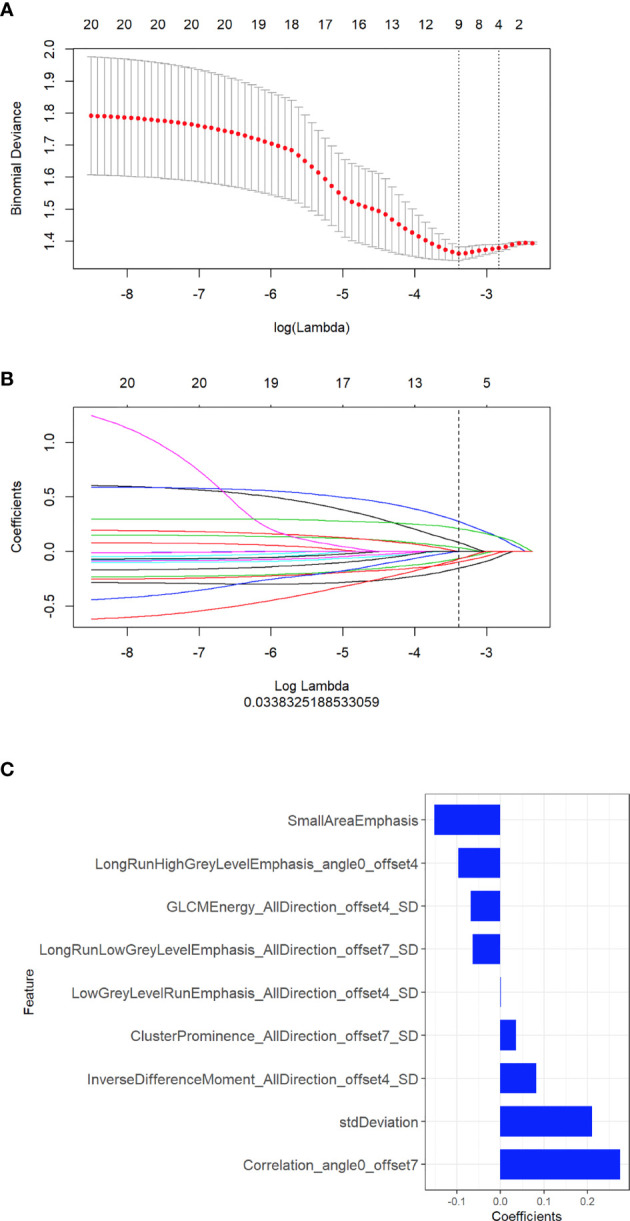
Selection of radiomic features associated with EGFR-activating mutations using the LASSO regression model. **(A)** Cross-validation curve. An optimal log lambda (0.03) was selected, and 9 non-zero coefficients were chosen. **(B)** LASSO coefficient profiles of the 396 radiomic features against the deviance explained. **(C)** Histogram showing the contribution of the selected parameters with their regression coefficients in the signature construction.

**Figure 2:**
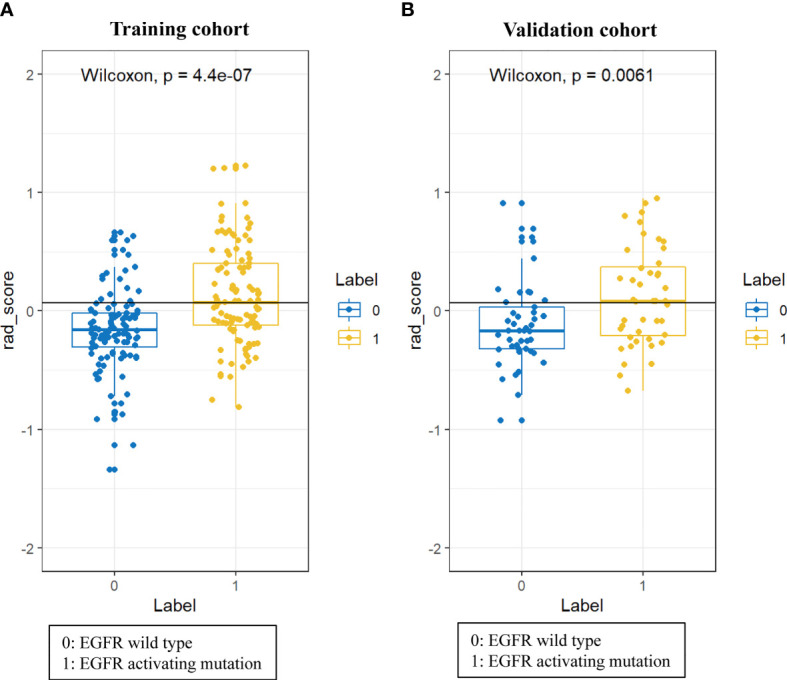
Difference in the Radscore between NSCLC patients with wild-type EGFR and EGFR-activating mutations in training cohort **(A)** and validation cohort **(B)**.

A total of 396 radiomic features were extracted from unenhanced CT images. The intraobserver ICCs ranged from 0.80 to 0.89, and the interobserver ICCs ranged from 0.76 to 0.90, indicating satisfactory intra- and interobserver feature extraction reproducibility. In all, 20 features were retained after the mRMR algorithm was applied. Then, LASSO was performed, including selection of the regular parameter λ (log λ=0.03), to determine the number of features (**Figures 1A, B**). After the number of features was determined, the most predictive subset of 9 features was chosen **(** **Supplementary Table 1** **)**, and the corresponding coefficients were evaluated (**Figure 1C**) and used to build a prediction model. The Radscore showed a significant difference between NSCLC patients with wild-type EGFR and EGFR-activating mutations in the training (P<0.0001) and validation cohorts (P=0.0061). Patients with EGFR-activating mutations generally showed a higher Radscore (**Figure 2**).

**Figure 3:**
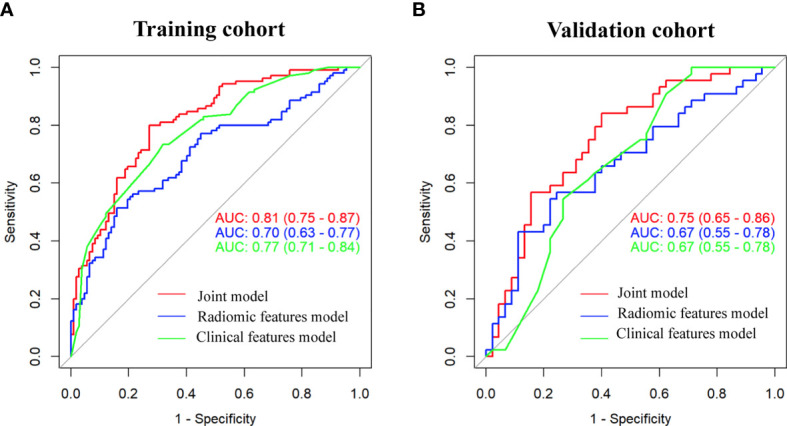
Comparison of performance among the three developed models for the prediction of EGFR-activating mutations in NSCLC patients. ROC curves of clinical features alone, radiomic features alone and combined features in the training **(A)** and validation **(B)** cohorts.

As shown in **Figure 3**, the radiomic feature only model achieved an AUC of 0.70 in the training cohort and 0.67 in the validation cohort. We incorporated the clinical indicators with P values less than 0.01 and the radiomic features into the logistic regression analysis (**Supplementary Table 2**). The joint model yielded an AUC of 0.81 (95% CI, 0.75-0.87) with a sensitivity of 84% in the training cohort (**Figure 3A**) and an AUC of 0.75 (95% CI, 0.65-0.86) with a sensitivity of 76% in the validation cohort (**Figure 3B**), which showed an improved performance over the radiomic signature in both the training and validation cohorts. **Table 2** lists the predictive performance of the joint model, using the AUC, accuracy, sensitivity and specificity as the main measurements. The joint model outperformed the radiomic feature model and the clinical characteristics-based model in terms of sensitivity in the training and validation cohorts.

**Table 2:** Predictive performance of the three models in the training and validation cohorts.

Model	Accuracy [95%CI]	AUC [95%CI]	Sensitivity	Specificity	P value
**Training cohort**					
Radiomic features	0.76 [0.70-0.82]	0.70 [0.63-0.77]	0.74	0.79	P < 0.0001
Clinical features	0.71 [0.64-0.77]	0.77 [0.71-0.84]	0.69	0.72	P < 0.0001
Joint features	0.68 [0.61-0.74]	0.81 [0.75-0.87]	0.84	0.51	P < 0.0001
**Validation cohort**					
Radiomic features	0.72 [0.60-0.80]	0.67 [0.55-0.78]	0.67	0.79	P = 0.0038
Clinical features	0.63 [0.52-0.73]	0.67 [0.55-0.78]	0.62	0.64	P = 0.0043
Joint features	0.66 [0.55-0.76]	0.75 [0.65-0.86]	0.76	0.57	P < 0.0001

AUC, Area under the curve; 95%CI, Confidence interval. The P value marked bold indicated statistical significance.

**Figure 4:**
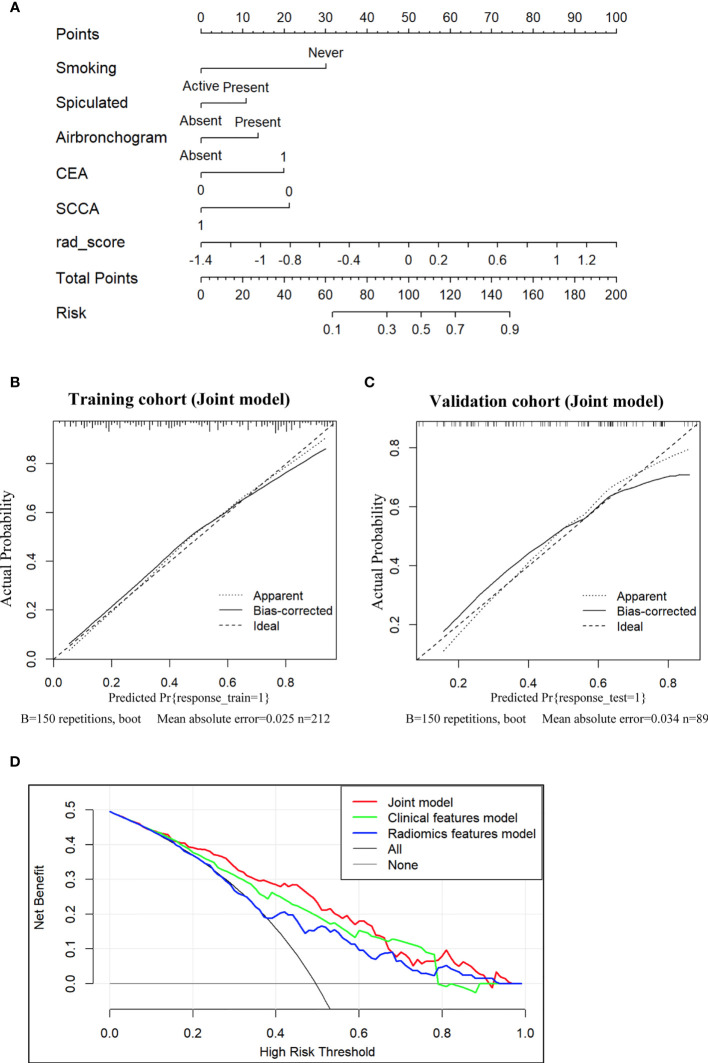
Nomogram for the prediction of EGFR-activating mutations based on the training cohort and the calibration curve for model evaluation. **(A)** Radiomic nomogram constructed with the clinical characteristics and Radscore. Calibration curves were used to assess the consistency between the nomogram-predicted EGFR-activating mutation probability and the actual fraction of EGFR-activating mutations in both the training **(B)** and validation **(C)** cohorts **(D)**. DCA for the prediction of EGFR-activating mutations in NSCLC patients for each model. The X-axis represents the threshold probability, and the Y-axis represents the net benefit. The net benefit is calculated by adding the benefits (true-positive results) and subtracting the risks (false-positive results), with the latter weighted by a factor related to the harm of an undetected cancer relative to the harm of unnecessary treatment. The red curve indicates the nomogram, which represents the joint prediction model composed of radiomic features and clinical indicators. The green curve represents the clinical feature model, while the blue curve represents the radiomic feature model. Our joint prediction model outperformed both the other models and simple strategies, such as the follow-up of all patients (grey line) or no patients (horizontal black line), across the majority of the range of threshold probabilities at which a patient would choose to undergo a follow-up imaging examination.

Subsequently, a nomogram integrating smoking status, spiculation, air bronchogram, CEA, SCCA and Radscore was constructed, as presented in **Figure 4A**. The calibration curve of the nomogram for the prediction of EGFR-activating mutations demonstrated favorable agreement between estimation with the radiomic nomogram and actual observations. The p value obtained *via* the Hosmer-Lemeshow test for the predictive ability of the nomogram was 0.57 in the training cohort (**Figure 4B**) and 0.24 in the validation cohort (**Figure 4C**).

DCA for the prediction model showed that the joint nomogram had the highest net benefit compared with the clinical and radiomic feature models across the majority of the range of reasonable threshold probabilities (**Figure 4D** **)**. The decision curve showed that if the threshold probability of a patient was within the range from 10% to 65%, using the joint nomogram developed in our study to predict EGFR-activating mutations added more benefit than the treat-all-patients scheme or the treat-no-patients scheme.

## Discussion

We undertook this study to develop and validate a joint model-based nomogram for the preoperative individualized prediction of EGFR-activating mutations in NSCLC patients. The nomogram integrated 5 clinical features, i.e., smoking status, spiculation, air bronchogram, CEA, and SCCA, and 9 radiomic features. Our findings suggest that NSCLC patients could be classified as having EGFR-activating mutations or wild-type EGFR according to our nomogram, indicating that the nomogram could be used as a novel and user-friendly instrument for the better management of NSCLC patients. Moreover, this study provides a visualized explanation to help clinicians understand the prediction outcomes in terms of CT data.

Diagnosis of the EGFR mutational status on an individual basis is vital for defining personalized treatment strategies. EGFR mutation including the sensitivity (EGFR Del19 and L858R) and resistance mutation (EGFR T790M) to TKIs. Recently, researchers have been seeking novel approaches to replace or complement conventional molecular analysis in routine CT examinations. Wang et al. proposed an end-to-end deep learning model to predict the EGFR mutation status by preoperational CT scanning, with an AUC of 0.85 in a primary cohort (24). However, the developed model can only be used to distinguish patients with wild-type EGFR and EGFR mutations and cannot identify whether mutations are EGFR activating or drug resistant mutations. In addition, although the deep learning method is labor-saving since it does not require precise nodule segmentation (25), the accuracy of segmentation is controversial. Liu et al. collected 289 patients with surgically resected peripheral lung adenocarcinomas and extracted 219 radiomic features to predict the EGFR mutation status, with an AUC of 0.709 (26). The prediction model in our study, with an AUC of 0.81 in the training cohort, is more reliable and can be used for discriminating wild-type EGFR and EGFR-activating mutations to guide targeted therapy.

Although smoking has been well established as the major cause of lung cancer, EGFR mutations have proved to be the most common genetic alteration in never-smoking NSCLC patients. A meta-analysis performed by Ren et al. revealed that non-smoking was associated with a significantly higher EGFR mutation rate. The frequency of EGFR mutations ranged from 22.7% to as high as 72.1% in never-smokers (27). Our results are in line with those of a previous study in that the presence of EGFR mutations was closely associated with the never-smoking status in NSCLC patients (28).

The relevance of CT features to the EGFR mutation status has also been reported recently. Spiculated margins, subsolid density, and non-smoking were confirmed to be significantly associated with EGFR-activating mutations (29). Zhou et al. found that spiculated margins, pleural retraction, and air bronchogram were more frequent in the EGFR mutation group than in the wild-type group, but there was no significant difference between these groups (30). On the other hand, air bronchogram was reported as an indicator of EGFR mutations in NSCLC (31). This result is consistent with Liu’s findings, which revealed a significant correlation between a small lesion size and air bronchogram with EGFR mutations in lung adenocarcinoma (32).

Serum tumor markers, such as CEA, SCCA, CYFRA 21-1, NSE, and ProGRP, are considered to be predictive or prognostic in NSCLC, and some of these markers have been shown to be correlated with EGFR mutations (33). CEA is widely known as a serum tumor marker of NSCLC (34, 35). It has also been uncovered that the serum CEA level in Chinese patients is not only positively associated with EGFR mutation but also negatively associated with the efficacy of TKI therapy (36). These findings raise the question of whether there is any correlation between the serum CEA level and EGFR mutations. In our study, the CEA level (below or above 5 ng/mL) served as an independent marker for predicting EGFR-activating mutations in NSCLC patients. Consistent with a previous report, an elevated serum CEA level predicted the presence of EGFR mutations in pulmonary adenocarcinoma (37). The low frequency of an elevated SCCA level has been reported in EGFR-mutated NSCLC, but no further evidence has been presented regarding the relation between SCCA and EGFR-activating mutations (38, 39). In our study, patients with a normal SCCA level showed higher scores, suggesting that this factor may contribute to the increased possibility of EGFR-activating mutations.

With the radiomic approach, we identified that 9 radiomic features from 4 different feature categories (GLCM, histogram, RLM, GLZSM) were significantly associated with EGFR-activating mutations and could serve as indicators for the prediction of EGFR-activating mutations. The AUC of the radiomic feature model was lower than that of the joint model (P=0.0005), suggesting that the radiomic features helped improve the performance of the joint model, as indicated by the higher AUC. These findings suggest that models integrating radiomic features with clinical features are more effective.

DCA demonstrated that the joint nomogram was superior to both the clinical feature model and the radiomic model across the majority of the range of reasonable threshold probabilities, which also indicates that the radiomic signature added value to the traditional clinical features used for individualized EGFR-activating mutation estimation. Therefore, a non-smoking patient presenting with an abnormal serum CEA level, a normal SCCA level, spiculation, air bronchogram and a high Radscore might be more likely to have EGFR-activating mutations.

This study has several limitations. First, this was a retrospective study and thus may have selection bias. Second, tumor segmentation was performed by a semi-automatic process, which was time consuming for the radiologists. However, the results are more robust, especially for tumors with unclear margins. Third, different CT scanning devices with different acquisition protocols were used. Thus, multicenter validation need to be performed to prove nomogram reliability.

In conclusion, we established a CT image-based model combining radiomic features and clinical variables for the prediction of EGFR-activating mutations before initial treatment in patients with NSCLC. The radiomic feature-based nomogram can serve as an alternative approach to determine better candidates for first-generation EGFR TKI therapy.

## Data Availability Statement

The raw data supporting the conclusions of this article will be made available by the authors, without undue reservation.

## Ethics Statement

The studies involving human participants were reviewed and approved by institutional review board of Lishui Hospital of Zhejiang University. The patients/participants provided their written informed consent to participate in this study.

## Author Contributions

QW and JJ designed the research. MX, WZ, JT and WM helped collect patient information. JuH, HW, and CLu performed experiments. JiH, CZ, PP, and LZ analyzed data. SF, MC, CLu, and YB prepared figures and tables. QW and JuH wrote the paper. HY and JJ conceived the project and supervised and coordinated all aspects of the work. All authors contributed to the article and approved the submitted version.

## Funding

This study was supported by Zhejiang Medical and Health Science Project (2019RC320 to QW, 2020KY1080 to HW, 2018KY197 to LZ, 2018KY932 to SF, 2018KY183 to YB), Natural Science Foundation of Zhejiang Province (LY18H160059 to JSH, LYY19H310004 to YB), The Public Welfare Project of Zhejiang Province (LGF18H160035 to HW), and the Science and Technology Project of Lishui City (2020ZDYF09 to QW). 

## Conflict of Interest

Author PP was employed by the company GE Healthcare Life Sciences (China).

The remaining authors declare that the research was conducted in the absence of any commercial or financial relationships that could be construed as a potential conflict of interest.

## Publisher’s Note

All claims expressed in this article are solely those of the authors and do not necessarily represent those of their affiliated organizations, or those of the publisher, the editors and the reviewers. Any product that may be evaluated in this article, or claim that may be made by its manufacturer, is not guaranteed or endorsed by the publisher.

## Abbreviations

ADC, Adenocarcinoma; AUC, Area under the curve; CEA, Carcinoembryonic antigen; CT, Computed tomography; CYFRA21-1, Cytokeratin 19-fragment; DCA, Decision curve analysis; EGFR, Epidermal growth factor receptor; EGFR DEL19, EGFR exon 19 deletions; EGFR L858R, EGFR exon 21 substitutions; GLCM, Gray-Level Co-occurrence Matrix; GLZSM=Gray level zone size matrices; LASSO, The least absolute shrinkage and selection operator; mRMR, The maximum relevance minimum redundancy; NSCLC, Non-small cell lung cancer; NSE, Neuron specific enolase; PCR, Polymerase chain reaction; PFS, Progression free survival; ProGRP, Progastrin-releasing peptide; Radscore, Radiomic scores; RLM, Run-length matrix; ROC, Receiver operating characteristic; ROI, Region of interest; SCC, Squamous cell carcinoma; SCCA, Squamous cell carcinoma antigen; TKIs, Tyrosine kinase inhibitors; 95%CI, 95% Confidence interval.

## References

[1] Chen S Feng S Wei J Liu F Li B Li X. Pretreatment Prediction of Immunoscore in Hepatocellular Cancer: A Radiomics-Based Clinical Model Based on Gd-EOB-DTPA-Enhanced MRI Imaging. Eur Radiol (2019) 29(8):4177–87. 10.1007/s00330-018-5986-x 30666445

[2] Cho A Hur J Moon YW Hong SR Suh YJ Kim YJ. Correlation Between EGFR Gene Mutation, Cytologic Tumor Markers, 18F-FDG Uptake in Non-Small Cell Lung Cancer. BMC Cancer (2016) 16:224. 10.1186/s12885-016-2251-z 26979333 PMC4793740

[3] Dagogo-Jack I Shaw AT. Tumour Heterogeneity and Resistance to Cancer Therapies. Nat Rev Clin Oncol (2018) 15(2):81–94. 10.1038/nrclinonc.2017.166 29115304

[4] Dal Bello MG Filiberti RA Alama A Orengo AM Mussap M Coco S. The Role of CEA, CYFRA21-1 and NSE in Monitoring Tumor Response to Nivolumab in Advanced Non-Small Cell Lung Cancer (NSCLC) Patients. J Transl Med (2019) 17(1):74. 10.1186/s12967-019-1828-0 30849967 PMC6408784

[5] Gao Y Song P Li H Jia H Zhang B. Elevated Serum CEA Levels Are Associated With the Explosive Progression of Lung Adenocarcinoma Harboring EGFR Mutations. BMC Cancer (2017) 17(1):484. 10.1186/s12885-017-3474-3 28705152 PMC5512835

[6] Garcia-Figueiras R Baleato-Gonzalez S Padhani AR Luna-Alcala A Vallejo-Casas JA Sala E. How Clinical Imaging Can Assess Cancer Biology. Insights Imaging (2019) 10(1):28. 10.1186/s13244-019-0703-0 30830470 PMC6399375

[7] Gillies RJ Kinahan PE Hricak H. Radiomics: Images Are More Than Pictures, They Are Data. Radiology (2016) 278(2):563–77. 10.1148/radiol.2015151169 PMC4734157 26579733

[8] Jiang R Wang X Li K. Predictive and Prognostic Value of Preoperative Serum Tumor Markers Is EGFR Mutation-Specific in Resectable Non-Small-Cell Lung Cancer. Oncotarget (2016) 7(18):26823–36. 10.18632/oncotarget.8662 PMC5042017 27072585

[9] Jin B Dong Y Wang HM Huang JS Han BH. Correlation Between Serum CEA Levels and EGFR Mutations in Chinese Nonsmokers With Lung Adenocarcinoma. Acta Pharmacol Sin (2014) 35(3):373–80. 10.1038/aps.2013.164 PMC4647899 24487967

[10] Kawaguchi T Ando M Asami K Okano Y Fukuda M Nakagawa H. Randomized Phase III Trial of Erlotinib *Versus* Docetaxel as Second- or Third-Line Therapy in Patients With Advanced Non-Small-Cell Lung Cancer: Docetaxel and Erlotinib Lung Cancer Trial (Delta). J Clin Oncol (2014) 32(18):1902–8. 10.1200/JCO.2013.52.4694 24841974

[11] Lambin P Leijenaar RTH Deist TM Peerlings J de Jong EEC van Timmeren J. Radiomics: The Bridge Between Medical Imaging and Personalized Medicine. Nat Rev Clin Oncol (2017) 14(12):749–62. 10.1038/nrclinonc.2017.141 28975929

[12] LeBleu VS. Imaging the Tumor Microenvironment. Cancer J (2015) 21(3):174–8. 10.1097/PPO.0000000000000118 PMC4498162 26049696

[13] Lee CK Davies L Wu YL Mitsudomi T Inoue A Rosell R. Gefitinib or Erlotinib *vs* Chemotherapy for EGFR Mutation-Positive Lung Cancer: Individual Patient Data Meta-Analysis of Overall Survival. J Natl Cancer Inst (2017) 109(6):1–9. 10.1093/jnci/djw279 28376144

[14] Lee CK Wu YL Ding PN Lord SJ Inoue A Zhou C. Impact of Specific Epidermal Growth Factor Receptor (EGFR) Mutations and Clinical Characteristics on Outcomes After Treatment With EGFR Tyrosine Kinase Inhibitors *Versus* Chemotherapy in EGFR-Mutant Lung Cancer: A Meta-Analysis. J Clin Oncol (2015) 33(17):1958–65. 10.1200/JCO.2014.58.1736 25897154

[15] Lee DH. Treatments for EGFR-Mutant Non-Small Cell Lung Cancer (NSCLC): The Road to a Success, Paved With Failures. Pharmacol Ther (2017) 174:1–21. 10.1016/j.pharmthera.2017.02.001 28167215

[16] Lin XF Wang XD Sun DQ Li Z Bai Y. High Serum CEA and CYFRA21-1 Levels After a Two-Cycle Adjuvant Chemotherapy for NSCLC: Possible Poor Prognostic Factors. Cancer Biol Med (2012) 9(4):270–3. 10.7497/j.issn.2095-3941.2012.04.009 PMC3643679 23691489

[17] Liu H Zhang C Wang L Luo R Li J Zheng H. MRI Radiomics Analysis for Predicting Preoperative Synchronous Distant Metastasis in Patients With Rectal Cancer. Eur Radiol (2019a) 29(8):4418–26. 10.1007/s00330-018-5802-7 30413955

[18] Liu Y Kim J Balagurunathan Y Li Q Garcia AL Stringfield O. Radiomic Features Are Associated With EGFR Mutation Status in Lung Adenocarcinomas. Clin Lung Cancer (2016a) 17(5):441–8.e6. 10.1016/j.cllc.2016.02.001 PMC5548419 27017476

[19] Liu Y Kim J Qu F Liu S Wang H Balagurunathan Y. Ct Features Associated With Epidermal Growth Factor Receptor Mutation Status in Patients With Lung Adenocarcinoma. Radiology (2016b) 280(1):271–80. 10.1148/radiol.2016151455 PMC4934516 26937803

[20] Liu Z Wang S Dong D Wei J Fang C Zhou X. The Applications of Radiomics in Precision Diagnosis and Treatment of Oncology: Opportunities and Challenges. Theranostics (2019b) 9(5):1303–22. 10.7150/thno.30309 PMC6401507 30867832

[21] Lu W Cham MD Qi L Wang J Tang W Li X. The Impact of Chemotherapy on Persistent Ground-Glass Nodules in Patients With Lung Adenocarcinoma. J Thorac Dis (2017) 9(11):4743–9. 10.21037/jtd.2017.10.50 PMC5720993 29268545

[22] Meurer WJ Tolles J. Logistic Regression Diagnostics: Understanding How Well a Model Predicts Outcomes. JAMA (2017) 317(10):1068–9. 10.1001/jama.2016.20441 28291878

[23] Reckamp KL Melnikova VO Karlovich C Sequist LV Camidge DR Wakelee H. and Quantitative Test Platform for Detection of NSCLC EGFR Mutations in Urine and Plasma. J Thorac Oncol (2016) 11(10):1690–700. 10.1016/j.jtho.2016.05.035 27468937

[24] Ren JH He WS Yan GL Jin M Yang KY Wu G. EGFR Mutations in Non-Small-Cell Lung Cancer Among Smokers and Non-Smokers: A Meta-Analysis. Environ Mol Mutagen (2012) 53(1):78–82. 10.1002/em.20680 22223435

[25] Rizzo S Petrella F Buscarino V De Maria F Raimondi S Barberis M. Ct Radiogenomic Characterization of EGFR, K-RAS, and ALK Mutations in Non-Small Cell Lung Cancer. Eur Radiol (2016) 26(1):32–42. 10.1007/s00330-015-3814-0 25956936

[26] Shi Z Zheng X Shi R Song C Yang R Zhang Q. Radiological and Clinical Features Associated With Epidermal Growth Factor Receptor Mutation Status of Exon 19 and 21 in Lung Adenocarcinoma. Sci Rep (2017) 7(1):364. 10.1038/s41598-017-00511-2 28336963 PMC5428650

[27] Soria JC Ohe Y Vansteenkiste J Reungwetwattana T Chewaskulyong B Lee KH. Osimertinib in Untreated EGFR-Mutated Advanced Non-Small-Cell Lung Cancer. N Engl J Med (2018) 378(2):113–25. 10.1056/NEJMoa1713137 29151359

[28] Sun Y Li C Jin L Gao P Zhao W Ma W. Radiomics for Lung Adenocarcinoma Manifesting as Pure Ground-Glass Nodules: Invasive Prediction. Eur Radiol (2020) 30(7):3650–9. 10.1007/s00330-020-06776-y PMC7305264 32162003

[29] Takeda M Nakagawa K. First- and Second-Generation EGFR-Tkis Are All Replaced to Osimertinib in Chemo-Naive EGFR Mutation-Positive Non-Small Cell Lung Cancer? Int J Mol Sci (2019) 20(1):1–8. 10.3390/ijms20010146 PMC6337322 30609789

[30] Thawani R McLane M Beig N Ghose S Prasanna P Velcheti V. Radiomics and Radiogenomics in Lung Cancer: A Review for the Clinician. Lung Cancer (2018) 115:34–41. 10.1016/j.lungcan.2017.10.015 29290259

[31] Ueno T Toyooka S Suda K Soh J Yatabe Y Miyoshi S. Impact of Age on Epidermal Growth Factor Receptor Mutation in Lung Cancer. Lung Cancer (2012) 78(3):207–11. 10.1016/j.lungcan.2012.09.006 23036155

[32] Wang Q Li Q Mi R Ye H Zhang H Chen B. Radiomics Nomogram Building From Multiparametric MRI to Predict Grade in Patients With Glioma: A Cohort Study. J Magn Reson Imaging (2019a) 49(3):825–33. 10.1002/jmri.26265 30260592

[33] Wang S Shi J Ye Z Dong D Yu D Zhou M. Predicting EGFR Mutation Status in Lung Adenocarcinoma on Computed Tomography Image Using Deep Learning. Eur Respir J (2019b) 53(3):1–44. 10.1183/13993003.00986-2018 PMC6437603 30635290

[34] Wu S Zheng J Li Y Yu H Shi S Xie W. A Radiomics Nomogram for the Preoperative Prediction of Lymph Node Metastasis in Bladder Cancer. Clin Cancer Res (2017) 23(22):6904–11. 10.1158/1078-0432.CCR-17-1510 28874414

[35] Wu Y Liu H Shi X Song Y. Can EGFR Mutations in Plasma or Serum be Predictive Markers of Non-Small-Cell Lung Cancer? A Meta-Analysis. Lung Cancer (2015) 88(3):246–53. 10.1016/j.lungcan.2015.03.008 25837799

[36] Xu L Yang P Liang W Liu W Wang W Luo C. A Radiomics Approach Based on Support Vector Machine Using MR Images for Preoperative Lymph Node Status Evaluation in Intrahepatic Cholangiocarcinoma. Theranostics (2019) 9(18):5374–85. 10.7150/thno.34149 PMC6691572 31410221

[37] Yang X He J Wang J Li W Liu C Gao D. CT-Based Radiomics Signature for Differentiating Solitary Granulomatous Nodules From Solid Lung Adenocarcinoma. Lung Cancer (2018) 125:109–14. 10.1016/j.lungcan.2018.09.013 30429007

[38] Zhao W Yang J Ni B Bi D Sun Y Xu M. Toward Automatic Prediction of EGFR Mutation Status in Pulmonary Adenocarcinoma With 3D Deep Learning. Cancer Med (2019) 8(7):3532–43. 10.1002/cam4.2233 PMC6601587 31074592

[39] Zhou JY Zheng J Yu ZF Xiao WB Zhao J Sun K. Comparative Analysis of Clinicoradiologic Characteristics of Lung Adenocarcinomas With ALK Rearrangements or EGFR Mutations. Eur Radiol (2015) 25(5):1257–66. 10.1007/s00330-014-3516-z 25577516

