# Supplementary material for: Radiomic Feature-Based Nomogram: A Novel Technique to Predict EGFR-Activating Mutations for EGFR Tyrosin Kinase Inhibitor Therapy
Source: Front Oncol. 2021 Aug 6;11:590937. doi: 10.3389/fonc.2021.590937 (PMC8377542; doi:10.3389/fonc.2021.590937)

Supplementary Material

| **Table S1. The selected Radiomic features** | | | |
| --- | --- | --- | --- |
| **Feature Name** | **Feature Class** | **Description** | **Coefficients** |
| (Intercept) |  |  |  |
| Variance | Histogram | The average of the squared differences from the Mean | -0.018 |
| Standard Deviation | Histogram | Measures the amount of variation or dispersion from the Mean Value | 0.211 |
| Long Run High Grey Level Emphasis_angle0_offset4 | RLM | Measures the joint distribution of long run lengths with higher gray-level values | -0.097 |
| Low Grey Level Run Emphasis_AllDirection_offset4_SD | RLM | Measures the distribution of low gray-level values, with a higher value indicating a greater concentration of low gray-level values in the image | 0.000 |
| Long Run Low Grey Level Emphasis_AllDirection_offset7_SD | RLM | The numbers of runs with pixels of gray level i and run length j for a given direction θ | -0.064 |
| Small Area Emphasis | GLZSM | A measure of the distribution of small size zones, with a greater value indicative of more smaller size zones and more fine textures. | -0.152 |
| Cluster Prominence_AllDirection_offset7_SD | GLCM | A measure of the skewness and asymmetry of the GLCM | 0.035 |
| Inverse Difference Moment_AllDirection_offset4_SD | GLCM | A measure of the local homogeneity of an image | 0.082 |
| Correlation_angle0_offset7 | GLCM | Measures the similarity of the grey levels in neighboring pixels | 0.275 |
| Energy_AllDirection_offset4_SD | GLCM | A measure of the magnitude of voxel values in an image | -0.068 |

| **Table S2. Selected clinical features and Radscore** | | | | |
| --- | --- | --- | --- | --- |
| **Feature Name** | **Description** | **OR** | **95% CI** | **P value** |
| Intercept |  | 0.167 | 0.065-0.401 | 0.000 |
| Smoking status | Active/Never | 4.672 | 2.334-9.719 | 0.000 |
| Spiculated | Present/Absent | 1.748 | 0.879-3.533 | 0.114 |
| Air bronchogram | Present/Absent | 2.025 | 0.939-4.497 | 0.076 |
| CEA | Normal/Abnormal | 2.786 | 1.449-5.519 | 0.000 |
| SCCA | Normal/Abnormal | 0.337 | 0.128-0.831 | 0.022 |
| Radscore |  | 6.256 | 2.64-016.207 | 0.000 |

Note. - CEA=Carcinoembryonic antigen, SCCA=Squamous cell carcinoma antigen.

**Figure S1. Five categories of AK extracted features.**


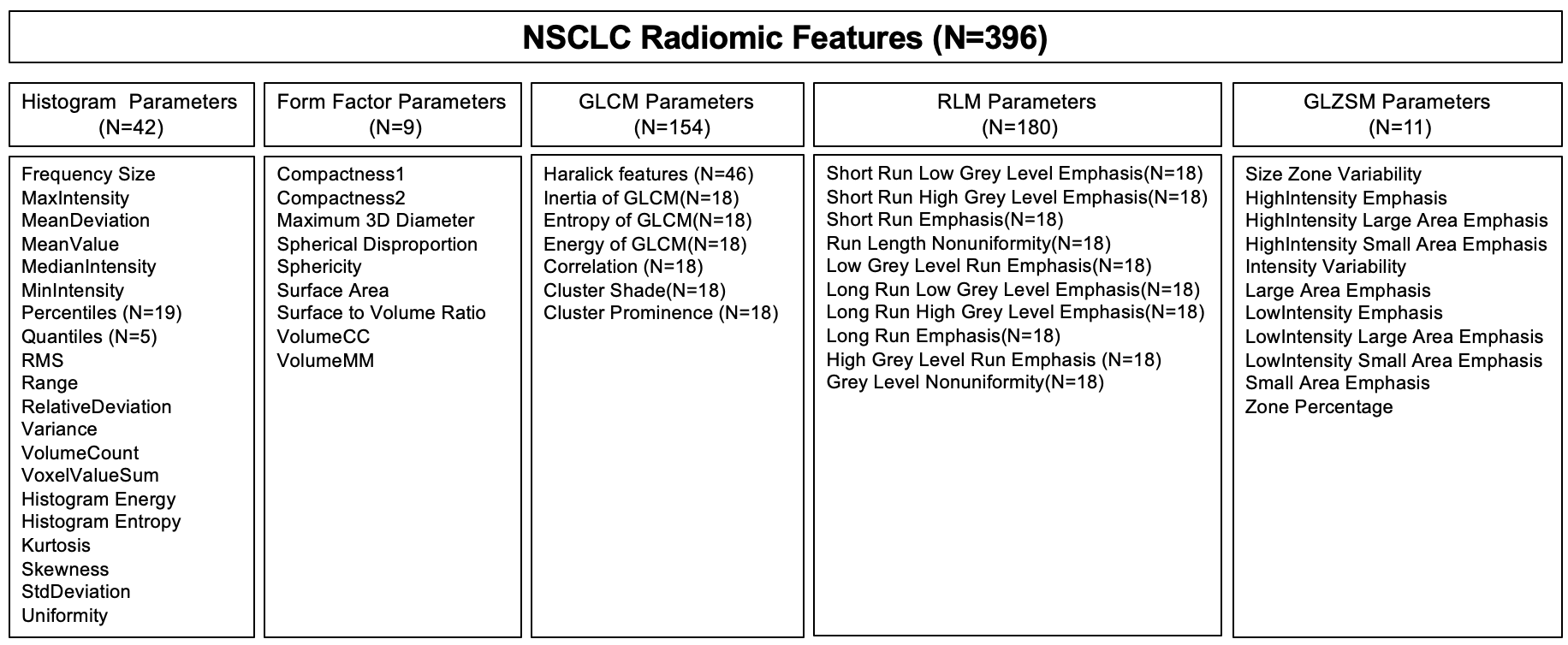

Supplement: Supplementary file 1 [file DataSheet_1.docx]
